# The Arbitrament of the Magistrate

**Published:** 1908-07-11

**Authors:** 


### Public Health and Hygiene.

### THE ARBITRAMENT OF THE MAGISTRATE.

It is a feature of public health administration in this country that the power to enforce even the simplest enactments is almost in all cases vested in the magistrate. Our magistrates do so much useful work, and on the whole do it so well, that criticism of them and of their work is pleasantly avoided. Nevertheless it is extremely questionable whether the training and habits of thought even of a stipendiary magistrate point to him as a suitable referee to decide in the administrative problems of public health, while it is the merest accident if a bench of magistrates is ever a proper tribunal for this purpose. The Poplar Borough Council has recently been moved to enter a dignified and reasonable protest against a magisterial decision, which well illustrates the unfitness of the magistrate to deal with the class of cases which arise in the administration of the Public Health Acts.

The landlord of certain houses in Poplar was summoned for a breach of by-laws made under Section 94 of the Public Health (London) Act, 1891. The Medical Officer of Health (Dr. Alexander) and the Sanitary Inspector gave evidence that certain rooms in the houses were in a "foul" and "filthy" condition, and in some cases were verminous. The magistrate, however, while agreeing that cleanliness would be promoted by the extermination of vermin, refused to make orders for cleansing or to inflict penalties upon the landlord for not causing rooms to be cleansed when they were made dirty through the wear and tear of the everyday life of the tenant. Although invited by the medical officer of health to view the premises, the magistrate refused either to accept the evidence as proof that the rooms were in a "foul and filthy" condition, or personally to satisfy himself upon this point by a visit to the premises.

Notwithstanding that no evidence was called to refute the evidence of the Council's officials, the summons was adjourned for a fortnight for the work of cleansing to be carried out.

Reporting on the matter, the Public Health Committee of the Borough Council state that the course taken by the magistrate appears to be highly unsatisfactory, and it is contended that he either should have dismissed the summonses, if the complaints were not well grounded, or inflicted penalties upon the defendant if the complaints were proved, as the by-laws are imperative. The Committee add:—

For a period of over fifteen years the medical officer of health has been giving evidence on behalf of the local authority in police courts and other courts, and if, according to the magistrate—who heard no rebutting evidence—the statements made by the medical officer of health in the witness-box were "grossly exaggerated," it is a matter for inquiry. For years the Public Health Department has been endeavouring to cause houses to be cleansed, and the by-laws were made for this purpose. Therefore it is regrettable that some magistrates do not support the local authority. If a magistrate will not make orders under the Public Health (London) Act in respect of dirty rooms being a nuisance or injurious or dangerous to health, and there is difficulty in obtaining penalties for not cleansing rooms under the by-laws for houses let in lodgings, perhaps if Mr. Burns's Housing, Town Planning, etc., Bill becomes law, Clause 27 will help local authorities in this most important matter. We are of opinion that in the interests of the efficient administration of the law relating to public health, it is necessary that notice should be taken of the course adopted by the magistrate on the proceedings in reference to these houses. If, after statutory notices have been served and all the attempts to induce owners to carry out their obligations have failed, summonses, taken as a last resource, are adjourned to enable the work to be done and then dismissed without penalties or costs, owners will be encouraged to resist the requirements of the Council on the chance of the failure of a prosecution, which, if successful, will cost them nothing, and the work of the Public Health Department will be much hampered. We deprecate also the remarks made by the magistrate on the evidence given on behalf of the Council, no evidence suggesting exaggeration having been offered on behalf of the defendant. We recommend that a copy of the report be forwarded to the Lord Chancellor, the President of the Board of Trade, and the Home Secretary.

In adopting the report, the Borough Council asked the Mayor, by a unanimous vote, to write a personal letter to accompany the report.

We quite agree with the Poplar Borough Council in the dignified attitude it has taken up. The main fault, of course, lies with the system. It is simply preposterous that an officer whose fitness to decide on questions of sanitation is the outcome of a long scientific training should be overruled by another public official whose training has not even aimed at qualifying him in this branch of knowledge. It is one of the chief merits of the Town Planning Bill that it eliminates the fantastic reference to the magistrate of questions relating to the habitableness of houses. This is so sound and is so daring an embodiment of common sense that only a cataclysm of precedent-ridden, tape-bound officialism could have permitted of it. We shall not despair of seeing substituted in time a tribunal of scientific men for the legal administrators of justice who at present invalidate the practical application of the findings of science.

